# A novel current steering method for targeted spinal cord stimulation

**DOI:** 10.3389/fpain.2023.1028368

**Published:** 2023-02-23

**Authors:** Lakshmi Narayan Mishra, Gaurav Kulkarni, Mandar Gadgil

**Affiliations:** ^1^ Nalu Medical Inc., Carlsbad, CA, United States; ^2^ Oneirix Engineering Laboratories Pvt. Ltd., Pune, MH, India

**Keywords:** multiple independent current sources, field focusing, current steering, neuromodulation, spinal cord stimulation, FEA

## Abstract

Spinal Cord Stimulation (SCS) with leads embedded in the epidural space has become a recognized and effective clinical therapy for chronic pain relief. Leads with multiple electrodes placed close to the spinal cord allow targeted stimulation. This paper presents a novel current steering method to achieve targeted spinal cord stimulation by determining the optimal current sourced through a set of electrodes to maximize current density in a specified region of the spinal cord. The method provides a flexibility for personalized pain relief therapy, while minimizing stimulation in unwanted regions. The paper proposes a new optimization problem to achieve current steering. The optimization problem uses a solution of the Poisson equation evaluated using Finite Element Analysis (FEA) over a geometric model of the spinal cord and the embedded leads. The solution to the optimization problem determines the optimal current sourced through a set of electrodes to achieve a targeted stimulation.

## 1. Introduction

Spinal cord stimulation (SCS) is a well-established technique for alleviating chronic pain. The Gate-Control theory implies that the effectiveness of pain relief depends on the selective activation of non-nociceptive nerve fibers within a nerve bundle (1, 2). Stimulation of nerve fibers leads to depolarization, which beyond a threshold, leads to generation of an action potential. Hence, to generate an action potential in selective non-nociceptive nerve fibers, targeted stimulation is necessary. Targeted stimulation not only improves the effectiveness of the stimulation but also may reduce power wastage by reducing the unwanted power dissipated in the other regions of the body.

Generating a localized electric field focused around the target region helps in achieving a targeted stimulation. Focusing the electric field in a region needs to be tuned based on the patient’s anatomy and the placement of electrodes relative to the body. This tuning can be achieved by specifying the current sourced through each implanted electrode. This method of achieving a targeted stimulation by controlling currents through the electrodes is called as current steering. Current steering systems could enable a better overlap of paresthesia and pain, which will have utility in clinical implementation of SCS. In previous studies (3, 4), different electrode placements have been tried to achieve a specific local electric field. Efforts have also been made to achieve a desired electric field by programmed excitation of fixed electrodes (5–7). Other studies (3, 8) have proposed different stimulation techniques for current steering, but a mathematical framework to determine the relative currents through the electrodes has not been presented.

Generation of an action potential in the desired nerve fibers due to targeted stimulation will be referred to as selective activation. Literature proposes various indicators to gauge the amount of nerve fibers activated. Selective activation has been indirectly monitored through the motor response in animals (4, 9). An approach of defining a selectivity index has also been tried (9). Activation has also been analyzed using mathematical models and by defining an activating function (10). Some recent computational models use a combination of Finite Element Analysis (FEA) for the electric field and a nerve model to gauge selective activation (5, 8, 11).

Activating function (10) estimates the generation of action potential in a nerve fiber by computing the second spatial derivative of the extracellular potential distribution along the nerve fiber axis. A closer look at its derivation shows that the activating function assists the flow of current at a node of Ranvier. The circuit representation considered by Rattay and the steps in the derivation are explained in more detail in the appendix section *Activating Function*. A positive value of the activating function indicates depolarization of the membrane. In order to achieve a positive activating function value, a relatively large amount of current should enter the stimulation target in comparison to its surroundings. In other words, the current density at the target location should be increased to achieve depolarization. Since the current density depends on the current sourced through the electrodes, the current through the electrodes should be optimally set to achieve targeted stimulation.

This paper presents a mathematical formulation of current steering as an optimization problem. The following section presents a computational model setup for obtaining the electrical field using Finite Element Analysis (FEA) and also describes the mathematical formulation for current steering. The further sections describe current steering in a situation where one or more of the stimulating electrodes are disconnected from the stimulation circuit. The paper also discusses a variant of the current steering problem where only one-sided current sources are available for stimulation. Results of the variants of the current steering method are discussed in the subsequent sections.

## 2. Methods

**Figure 1:**
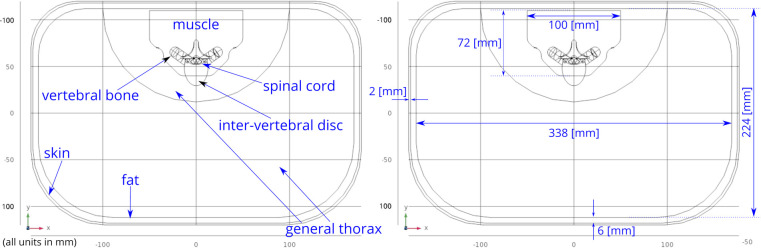
Abdomen cross-section. The general thorax section has been divided into two sections—the outer section has a coarser mesh, the inner section near the spinal cord has finer mesh. This division allows efficient meshing while resolving the electric field accurately.

**Figure 2:**
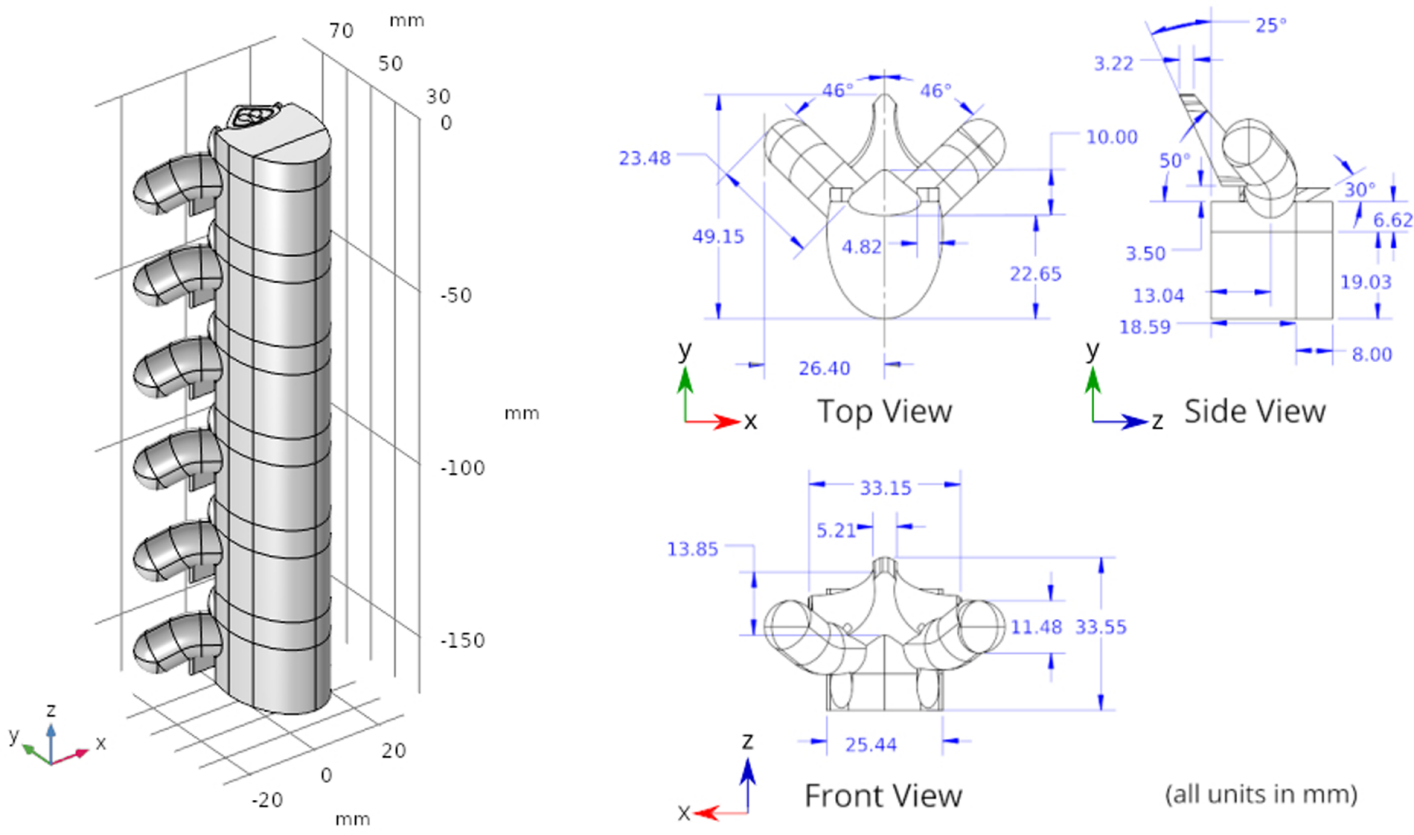
Spine geometry. The Z axis is aligned along the spinal cord. The XY plane is the transverse plane perpendicular to the spinal cord.

**Figure 3:**
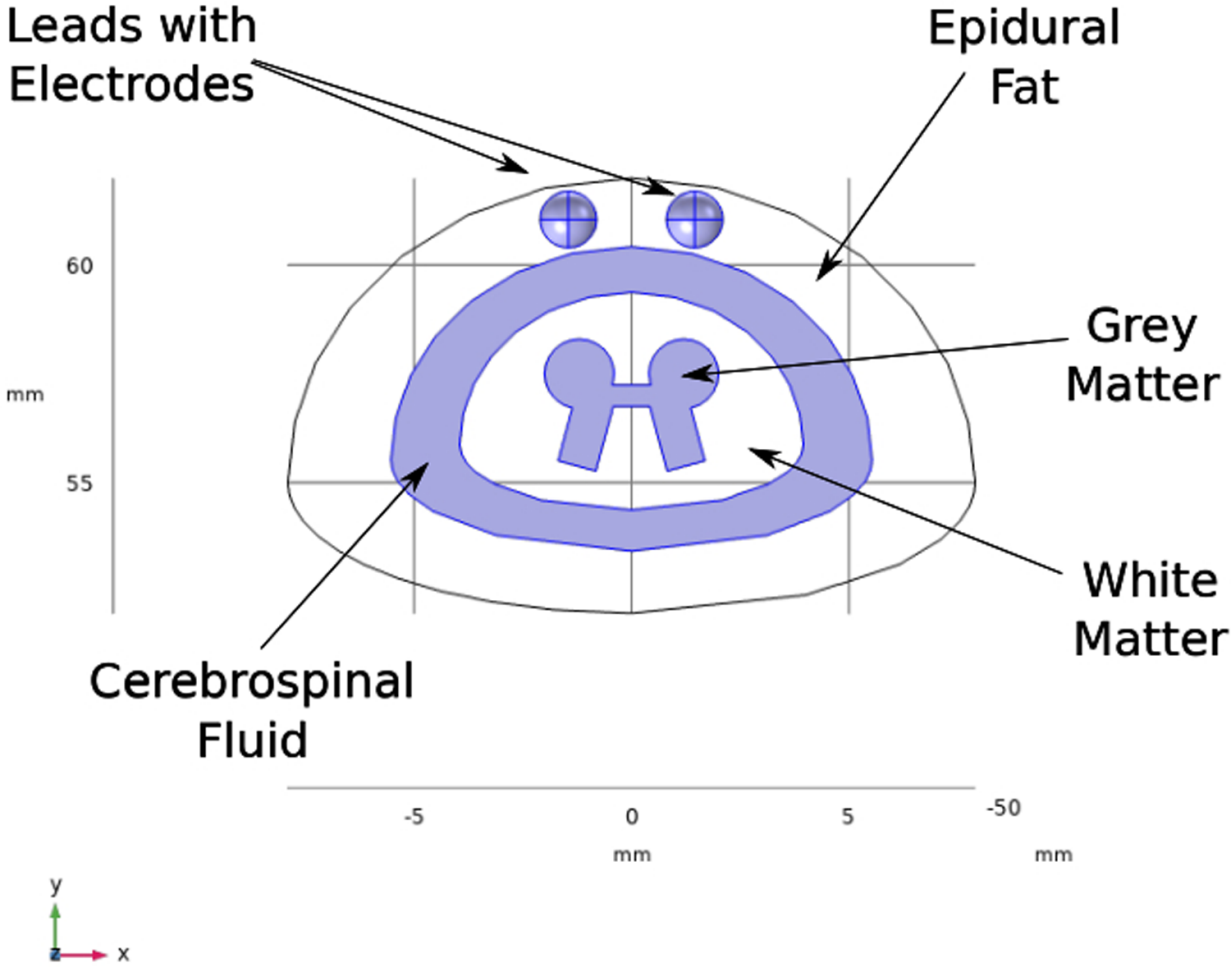
Cross-section of spinal cord. The leads are embedded in the epidural fat layer.

A three-dimensional computational model of the human body near the spinal cord region has been developed for calculating the electric field. This computational model shown in Figure 1, consists of a three-dimensional geometry of the human body near the spinal cord region, including the various components such as skin, fat, thorax, muscle, vertebral bones, inter-vertebral discs and the spinal cord. The geometry of the spinal cord shown in Figure 2, considers the anatomical details such as the epidural fat, cerebrospinal fluid, white matter and grey matter (12). Two implanted leads, each consisting of 8 electrodes, embedded in the epidural fat are also considered in the computational model as shown in Figure 3. COMSOL Multiphysics® (13), a finite element based software, is used to solve the differential equations governing the flow of electric charges given by the Poisson equation (1). The geometry is discretized using tetrahedral mesh elements. The spinal cord, muscle and the inner section of the general thorax near the spinal cord have a finer mesh (min. element size 0.5[mm] and max. element size 12[mm]). Other components have a relatively coarser mesh. This ensures better resolution of the electric field in the spinal cord domain while having a feasible number of mesh elements. This study performs quasi-static simulations to solve the Poisson’s equation (1) and obtain the electric field distribution. To solve the Poisson’s equation, the domain is discretized as a computational mesh and the material properties are assigned to each domain as listed in Table 1. The outermost skin boundary is specified an insulating boundary condition. Dirichlet boundary conditions for the electrodes are specified based on the electrode excitation pattern, as described in section “Optimization problem for current steering.”

**Table 1:** Material properties.

Component	Conductivity [S/m]
Grey matter	0.23
White matter	{0.083,0.083,0.6}
Cerebrospinal fluid	1.7
Epidural fat	0.04
Vertebral bone	0.02
Inter-vertebral disc	0.6
Muscle	{0.08,0.08,0.5}
General thorax	0.25
Skin	0.0025
Fat	0.04
Lead	$1\times 10^{-16}$
Contacts	$4\times 10^{6}$

The conductivity for white matter and muscles is not isotropic. A conductivity vector is considered to specify the conductivity along the X-Y-Z directions. For the geometry considered, the Z direction is along the spinal cord. The XY plane represents the transverse plane perpendicular to the spinal cord.

### 2.1. Optimization problem for current steering

The solution of the Poisson equation (1) gives the potential field $f_{\vec{x}}$ at each position $\vec{x}\equiv (x,y,z{)}^{T}$ in the complete domain Ω, which includes the spinal cord, thorax and other regions described in Figure 1.

(1) $$\nabla \cdot (\sigma_{\vec{x}}\nabla f_{\vec{x}})=0\in \Omega$$

where $\sigma_{\vec{x}}$ is the conductivity tensor at $\vec{x}\in \Omega$.

The aim of the optimization problem is to maximize the current density ($\|\sigma_{\vec{x}}\nabla f_{\vec{x}}{\|}_{2}$) in the target region $\Omega_{T}$ while minimizing the current density in the rest of the domain. Thus, the objective function takes the following form:

(2) $$\min_{\;f_{\vec{x}}}\frac{{∭}_{\Omega -\Omega_{T}}\|\sigma_{\vec{x}}\nabla f_{\vec{x}}{\|}_{2}^{2}\;dx\;dy\;dz}{{∭}_{\Omega_{T}}\|\sigma_{\vec{x}}\nabla f_{\vec{x}}{\|}_{2}^{2}\;dx\;dy\;dz}$$

The minimization problem defined in (2) maximizes the total norm square of the current density ${∭}_{\Omega_{T}}\|\sigma_{\vec{x}}\nabla f_{\vec{x}}{\|}_{2}^{2}\;dx\;dy\;dz$ in the target region and simultaneously minimizes the total norm square of the current density in the remaining domain ${∭}_{\Omega -\Omega_{T}}\|\sigma_{\vec{x}}\nabla f_{\vec{x}}{\|}_{2}^{2}\;dx\;dy\;dz$. It is important to minimize the current density in the remaining domain so as to avoid any unwanted stimulation. The detailed derivation of the formulation is given in the appendix section *Optimization Problem Formulation*. The final optimization problem turns out to be,

(3) $$\max_{v_{i}}\frac{V^{T}CV}{V^{T}C_{0}V}$$

and this problem can be represented as a constrained optimization problem as follows,

(4) $$\max_{v_{i}}\;V^{T}CV$$

(5) $$s.t.\;V^{T}C_{0}V=1$$

where,

(6) $$V=(v_{1}\;v_{2}\ldots v_{q}\ldots v_{N}{)}^{T}$$

are the voltages on each electrode and C and $C_{0}$ are square symmetric matrices computed from FEA simulation results. The solution to the optimization problem is detailed in appendix section *Solution to Quadratic Optimization Problem*. The solution is found by solving the following well known *generalized eigenvalue* problem (14).

(7) $$CV=\lambda C_{0}V$$

The maximum eigenvalue $\lambda^{*}$ is the maximum value of the objective function and the corresponding eigenvector is the optimal voltage vector $V^{*}$. The corresponding electrode currents $I^{*}$ can be calculated using the conductance matrix Y.

(8) I=YV

The conductance matrix defines a linear relationship between electrode current and voltage. It also ensures that the Kirchhoff’s current law, $\sum_{p}i_{p}=0$, where $i_{p}$ is p^th^ component of I, is satisfied. In a FEA simulation, if a Dirichlet boundary condition of 1[V] is specified for the q^th^ electrode and the remaining electrodes are grounded (Dirichlet boundary condition of 0[V]), then the current flowing through respective electrodes will represent the q^th^ column of the conductance matrix Y. As an example, consider a system with 3 electrodes. 3 simulations will be required in this case for determining the conductance matrix. In the first simulation, 1[V] voltage will be applied to the 1^st^ electrode and the remaining 2 electrodes will be grounded (0[V]). The current through the three electrodes will represent the 1^st^ column of the Y matrix as shown in Equation (9). Similarly, two more simulations will be performed with the 2^nd^ and 3^rd^ electrode given 1[V] respectively. In this way, conductance matrix Y can be computed through FEA by specifying appropriate voltages to the electrodes.

(9) $$\left[\begin{array}{ccc} Y_{11} & Y_{12} & Y_{13}\\ Y_{21} & Y_{22} & Y_{23}\\ Y_{31} & Y_{32} & Y_{33} \end{array}\right]\left[\begin{array}{c} 1\\ 0\\ 0 \end{array}\right]=\left[\begin{array}{c} Y_{11}\\ Y_{21}\\ Y_{31} \end{array}\right]=\left[\begin{array}{c} I_{1}\\ I_{2}\\ I_{3} \end{array}\right]$$

The optimal electrode currents $\left(I^{*}=YV^{*}\right)$ generate a potential field in the body such that it maximizes the current density in the target domain and simultaneously minimizes the current density in other domains. A positive value in the current vector implies that the electrode needs to act as an anodic current source whereas a negative value implies that the electrode has to act as a cathodic current source.

### 2.2. Current steering with disconnected electrodes

The current steering formulation presented above considers all electrodes from all the leads to achieve targeted stimulation. Sometimes an electrode may get disconnected from the stimulation circuit (such as open circuits due to lead wire breakage). This adversely affects targeted stimulation. In order to minimize its impact, it is important to find a relative current distribution among the working electrodes to achieve targeted stimulation. The optimization problem presented earlier can be modified to achieve targeted stimulation even when a set of electrodes are disconnected from the stimulation circuit.

Disconnected electrodes are floating i.e. no current flows through these electrodes. This adds a constraint to the optimization problem. Consider the linear current-voltage relation Equation (8), where the conductance matrix Y is a (N×N) square matrix where N is the number of electrodes. Since no current flows through the floating electrodes, entries in the current vector I corresponding to these electrodes is 0.

Let the rows of the conductance matrix Y be split into two sub-matrices, $Y_{w}$ for working electrodes and $Y_{f}$ for floating electrodes as follows,

(10) $$I_{w}=Y_{w}V$$

(11) $$0=Y_{f}V$$

Let m be the number of floating electrodes. Then $Y_{f}$ is a (m×N) dimensional matrix and $Y_{w}$ is a (N−m×N) dimensional matrix . Equations (10) and (11) specify the floating electrode constraint on V. Let a (N×N−m) dimensional basis matrix $B_{w}$, be such that it restricts the voltage vector V to a linear subspace spanned by $Y_{w}^{T}$ and orthogonal to $Y_{f}^{T}$ (Equations (10) and (11)). Q-R decomposition (15) can be used to find such a basis matrix $B_{w}$.

(12) $$V=B_{w}\alpha$$

where α is the vector of linear combiners of basis $B_{w}$.

Substituting Equation (12) in the optimization problem (3), we get,

(13) $$\max_{v_{i}}\frac{V^{T}CV}{V^{T}C_{0}V}=\max_{\alpha }\frac{\alpha^{T}B_{w}^{T}CB_{w}\alpha }{\alpha^{T}B_{w}^{T}C_{0}B_{w}\alpha }=\max_{v_{i}}\frac{\alpha^{T}D\alpha }{\alpha^{T}D_{0}\alpha }$$

where, $D=B_{w}^{T}CB_{w}$ and $D_{0}=B_{w}^{T}C_{0}B_{w}$.

Equation (13) has the same form as the original optimization and hence the generalized eigenvalue problem, $D\alpha =\lambda D_{0}\alpha$ gives the solution to this constrained optimization problem. The maximum eigenvalue $\left(\lambda^{*}\right)$ will be the maximum value of the objective function and the corresponding eigenvector $\alpha^{*}$ will represent the optimum value of the linear combiners such that the current through the floating electrodes is always zero. The optimal stimulation currents are:

(14) $$I^{*}=YB_{w}\alpha^{*}$$

### 2.3. Current steering using one-sided current sources

A stimulation electronic circuit having only one-sided current sources allows for a compact pulse generator size. A one-sided current source refers to a design that includes either current sources or current sinks but not both. The current sources and associated switches (that connect the sources/sinks to the electrodes) are generally the largest structures on the integrated circuit (IC) that constitute a neurostimulator. A current steering approach that accomplishes the task of focusing the current without needing both sources and sinks is therefore advantageous in maintaining a small IC and by extension a small implantable pulse generator (IPG).

The optimization problem (13) described above can be extended further to consider one-sided current sources. In specifying the optimization problem, among the working electrodes, one has to specify the set of ground electrodes (0[V]) and the set of source electrodes. Note that the current sinking through the ground electrodes depends on the anatomy and position of the ground electrodes. An optimization problems has to be solved for each combination of ground and source electrodes and the combination that gives the maximum objective function value has to be selected as a solution. Each combination should have at least one ground electrode and at least one current source to ensure Kirchhoff Current Law (KCL) is obeyed.

Let us assume that the q^th^ electrode is grounded i.e. $v_{q}=0[V]$. Then, substituting it in the optimization problem (3) reduces the dimensionality of the problem. It can be seen that with $v_{q}=0[V]$, q^th^ row and column of C and $C_{0}$ matrices do not contribute to the optimization problem. Hence the q^th^ row and column can be removed from those matrices to arrive at a reduced dimensionality generalized eigenvalue problem. The solution to this problem will always have 0[V] assigned to all the selected ground electrodes.

In order to allow only one-sided current sources, we extend the optimization problem (13) as,

(15) $$\max_{\alpha }\;\frac{\alpha^{T}D\alpha }{\alpha^{T}D_{0}\alpha }$$

(16) $$s.t.\;YB_{w}\alpha \geq 0$$

where, $D=B_{w}^{T}CB_{w}$, $D_{0}=B_{w}^{T}C_{0}B_{w}$ and $I=YB_{w}\alpha$ gives the currents sourced from each electrode.

The reduced dimensionality C and $C_{0}$ matrices are used to compute D, $D_{0}$ and $B_{w}$ matrices. The linear constraint $I=YB_{w}\alpha \geq 0$ specifies the constraint for one-sided anodic current sources. The above optimization problem is a linearly constrained generalized eigenvalue problem. Constraints like $I=YB_{w}\alpha \geq 0$ cannot be handled in a convex optimization framework. Hence, a locally optimal solution needs be found by using an iterative solver like Sequential Quadratic Programming (SQP) (16).

The selected ground electrodes and the distribution of current among the source electrodes (found by solving the optimization problem) together specifies the solution for current steering in the case of one-sided current sources.

### 2.4. Current steering method - clinical implementation

Three optimization problems are described above, corresponding to the implanted leads having two-sided current sources, one-sided current sources and disconnected electrodes. The solution of the optimization problems considers all electrodes present on the two leads as active electrodes. In order to reduce the computational complexity, maximum 8 electrodes (4 on each lead) that are nearest to the target location are considered for optimization. Any remaining electrodes are assumed to be floating $\left(I_{i}=0\right)$. For considering floating electrodes, the current steering formulation for disconnected electrodes is used.

**Figure 4:**
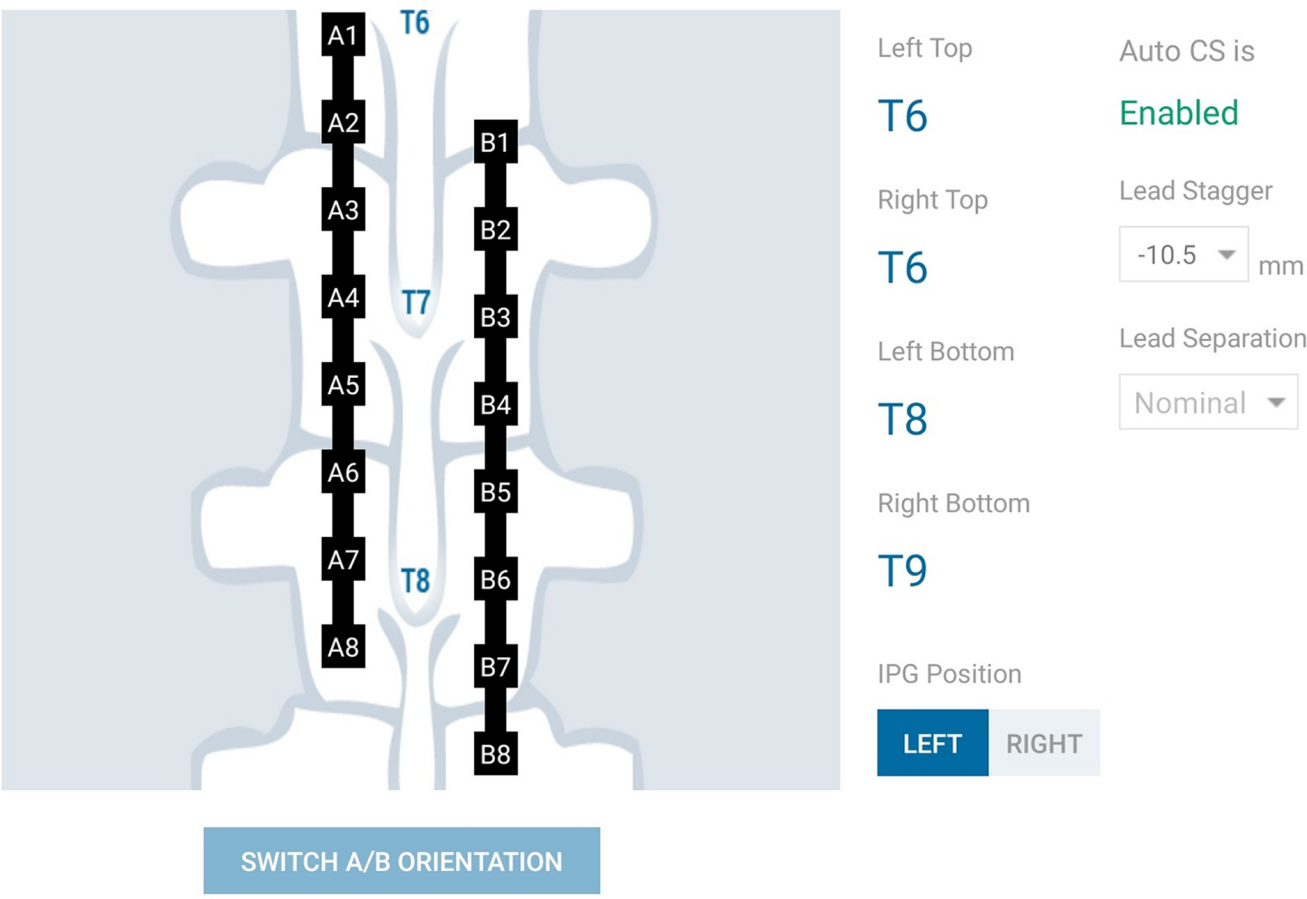
Schematic representation of lead configuration.

**Figure 5:**
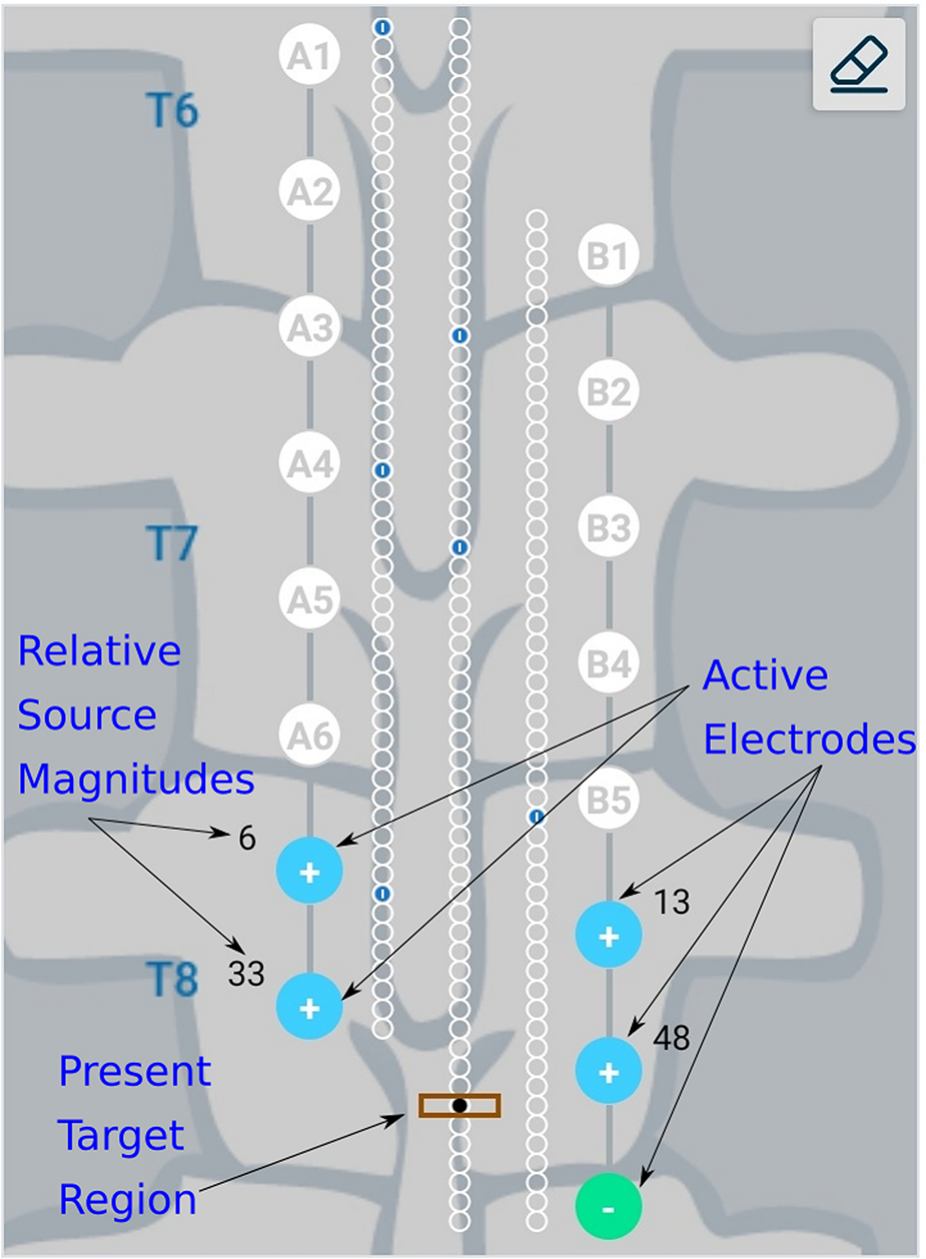
Schematic representation of user interface. Green, filled circles indicate current sinks and blue, filled circles indicate current sources. The numbers near the electrodes represent the relative percentage of current sourced/sunk through the electrodes. Small blue circles with a dash inside them represent target locations which have already been checked by the clinician.

The Nalu Neurostimulation System (Carlsbad, CA) implements the current steering method described above. The FEA model is solved for a multitude of lead placements and the resulting data is stored in a SQL (Structured Query Language) database. At run time the Clinician Programmer accesses the pre-computed data based on the user selection from the appropriate tables that correspond to the particular patients configuration. As shown in Figure 4, the clinician provides the lead configuration data (lead location, separation and offset). Lead configuration data is entered via a user interface that provides selections for the distance between the leads (in 3 settings) and the stagger (in steps of 0.5[mm]). Thereafter the clinician is provided with an interface shown in Figure 5 that allows them to select a focus location for the targeted stimulation. The interface shows the active electrodes and the relative percentage of source magnitudes (rounded off to the nearest integer) for the present target region. The interface also provides a visual history of the target regions selected previously. The clinician can rapidly switch between target regions to evaluate the best response for the patient. The optimum electrode excitation pattern and the system configuration can be downloaded to the patient device.

## 3. Results

**Figure 6:**
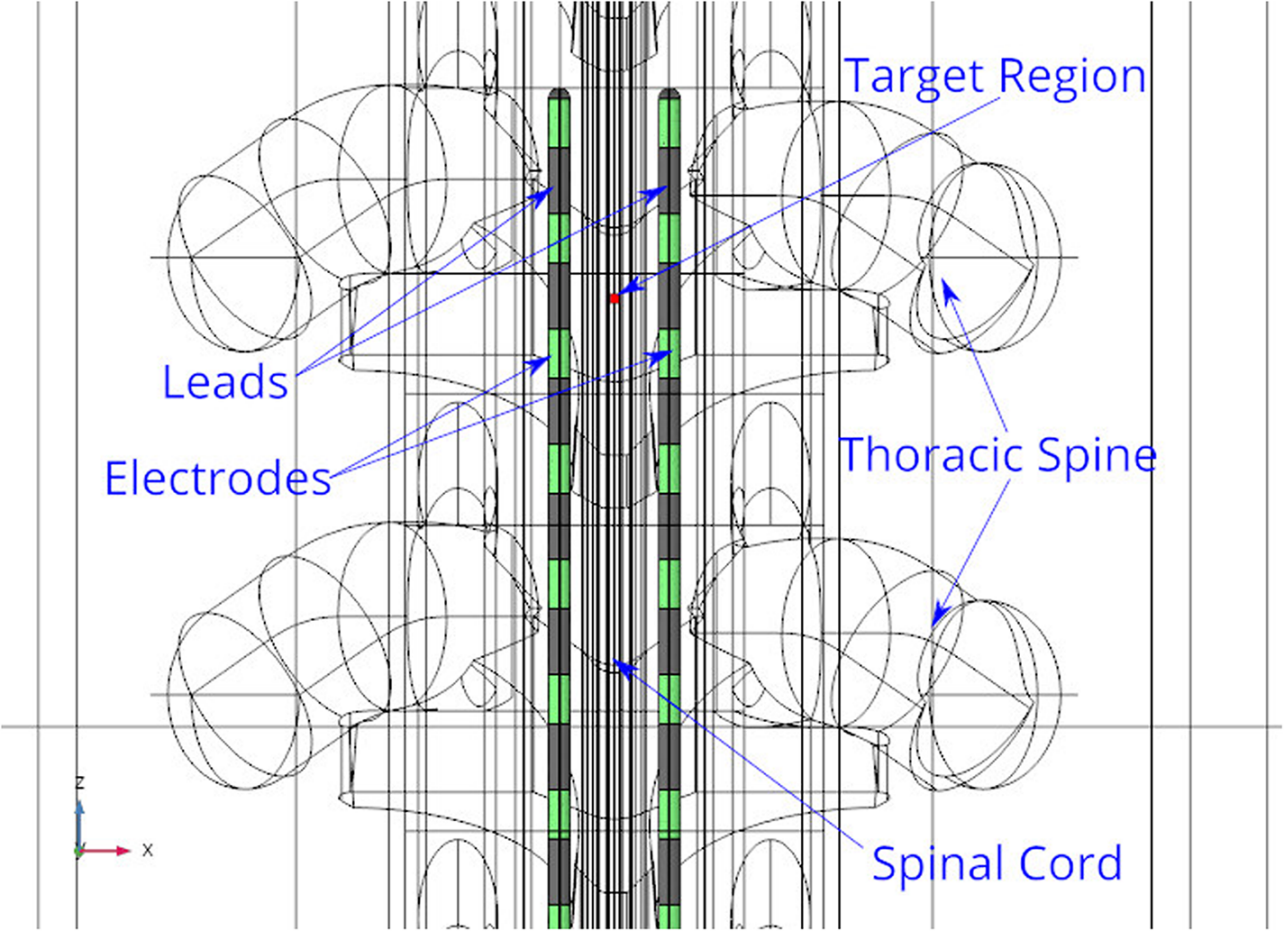
Stimulation leads and target region. The leads are shown in grey color and the electrodes are shown in green color. The target region shown in red color, is placed within the white and grey matter domains.

To define a target region for steering current, a cylindrical domain of 0.5[mm] diameter, approximately half the inter-nodal distance of nerve fiber, is specified. A parallel-lead geometry with the electrodes on the two leads aligned to each other, is considered for presenting the results. Figure 6 shows the top view of this geometry, with two leads placed in the spinal cord’s epidural fat region and the stimulation target in front of one of the electrodes.

**Figure 7:**
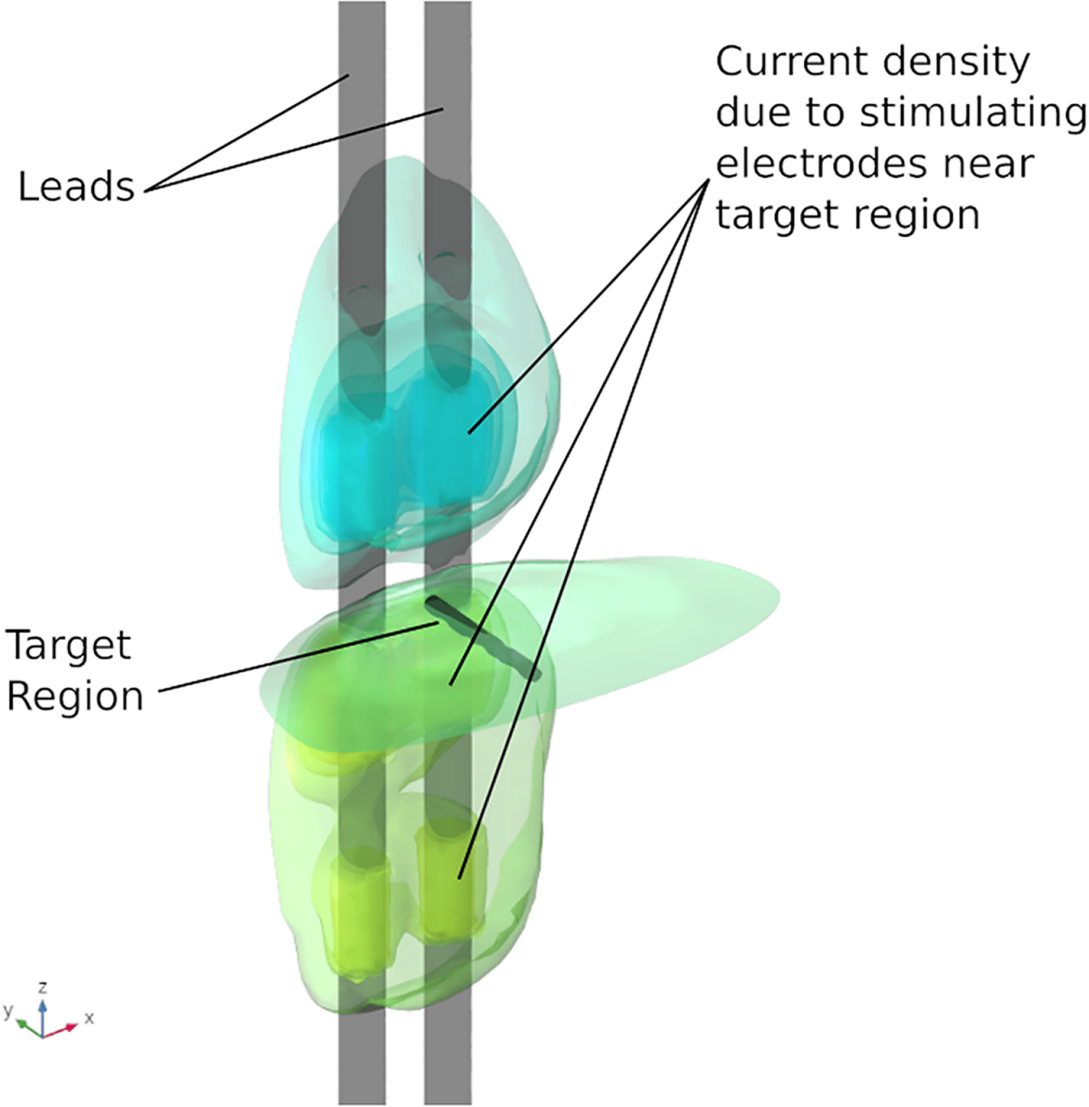
Current density near target region. The electrodes in the immediate vicinity of the target region are active.

**Figure 8:**
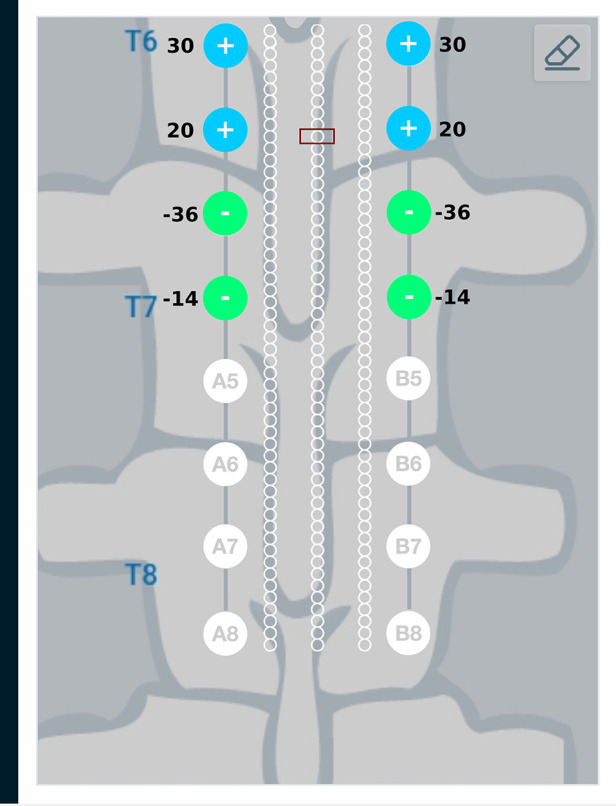
Current steering results graphically represented on the user interface. The current steering results are obtained in the form of the optimum electrode excitation pattern – shown on the parallel leads in this figure. Green, filled circles indicate current sinks and blue, filled circles indicate current sources. The numbers near the electrodes represent the relative percentage of current sourced/sunk through the electrodes.

Results from the finite element analysis (FEA) are post-processed to visualize the current density at the target region. The aim is to observe the current density in the target region when the optimal current $I^{*}$ (solution to optimization problem given in Equation (3)) is applied on the electrodes. The current density distribution near the target region is shown in Figure 7. For this target region, the solution to the optimization problem selected eight electrodes to steer the current. In the solution, two electrodes on each lead act as anodic current sources and the two electrodes on each lead act as cathodic current sources. It can be observed that all the selected electrodes are in the vicinity of the target region. For better visualization, the current steering results have also been graphically represented on the user interface, shown in Figure 8. The UI considers two leads parallel to each other and the electrodes aligned to each other. All the target regions considered in the FEA simulations are represented as white circles on the UI. A red rectangular box highlights the target region being considered for current steering. This target region shown in Figure 8 is located at an equal distance from both the leads. A green, filled circle indicates that the electrode is a current sink. A blue, filled circle indicates that the electrode is a current source. The numbers near the electrodes indicate the relative percentage of current (rounded off to the nearest integer) sourced/sunk through the electrode.

**Figure 9:**
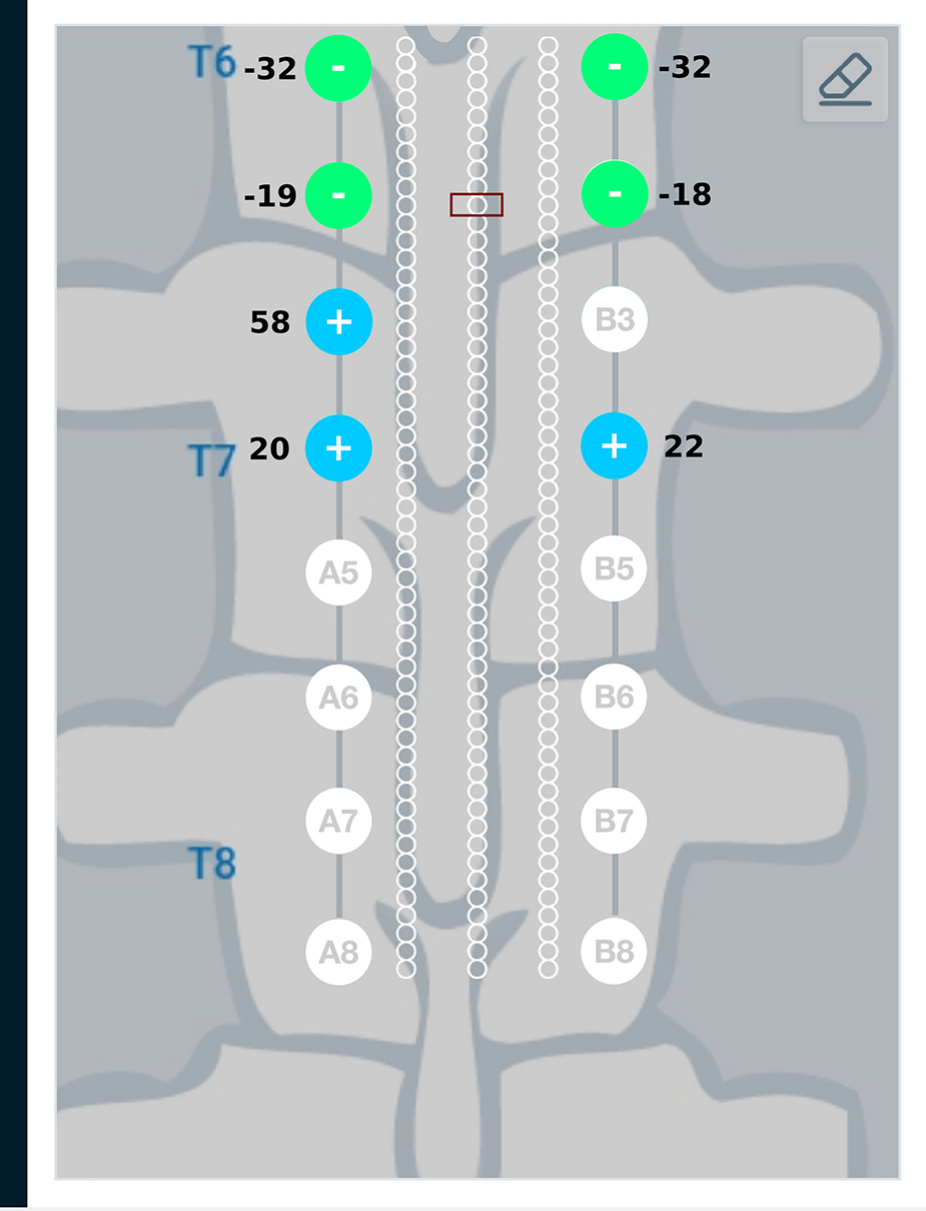
Current steering results graphically represented on the user interface. The current steering results are shown for 2-sided current sources with electrode B3 assumed to be disconnected—the current steering solution gives the optimum electrode excitation pattern without using B3 as an active electrode. In comparison to Figure 8, we can observe that more current is sourced from electrode A3 to compensate for B3 as a floating electrode.

Result from the current steering method considering disconnected electrodes (solution to the optimization problem given in Equation (14)) for the target region described above, are graphically represented in Figure 9. As an example, electrode B3—the 3rd electrode on the right lead—is considered to be disconnected from the circuit. This requires electrode B3 to be floating as described in the optimization problem. Figure 9 shows a difference in the optimum electrode excitation pattern as compared to Figure 8. Electrode A3 sources more current as compared to solution in Figure 8, so as to compensate for the disconnected electrode. Electrodes A4 and B4 also source relatively more current for the same reason.

**Figure 10:**
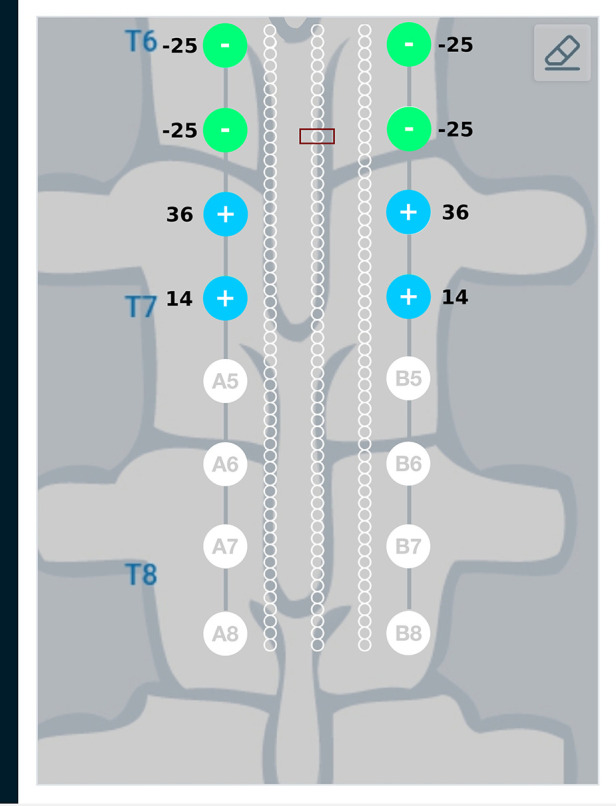
Current steering results graphically represented on the user interface. The current steering results are shown for one-sided current sources—the current steering solution gives the optimum electrode excitation pattern while specifying only the current sources. In comparison to Figure 8, we can observe that the source and sink electrodes have changed. Electrodes A3 and B3 which are closer to the target region source more current compared to electrodes A4 and B4. As the amount of current sinking cannot be specified, these amounts are governed by the patient anatomy, conductivity values of the domains and relative lead placements.

Figure 10 provides a graphical representation of results from the current steering method considering anodic current sources (solution to the optimization problem given in Equations (15) and (16)) for the target region described above. As seen in Figure 10, the source and sink electrodes have changed in comparison to Figure 8. Electrodes A3 and B3 which are closer to the target region, source more current as compared to electrodes A4 and B4. The amount of current sinking through electrodes A1, A2, B1, B2 depend on the patient anatomy, conductivity values of the domains and the relative placement of the leads.

## 4. Discussion

**Figure 11:**
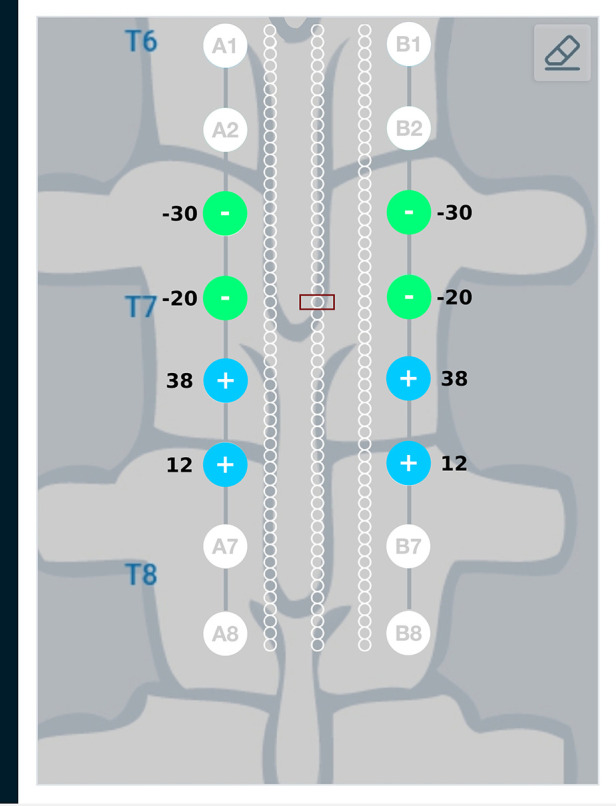
Current steering results graphically represented on the user interface. For the target region located at an equal distance from the two leads, the optimal electrode excitation shows that the current sourced/sunk from lead A and lead B is equal.

**Figure 12:**
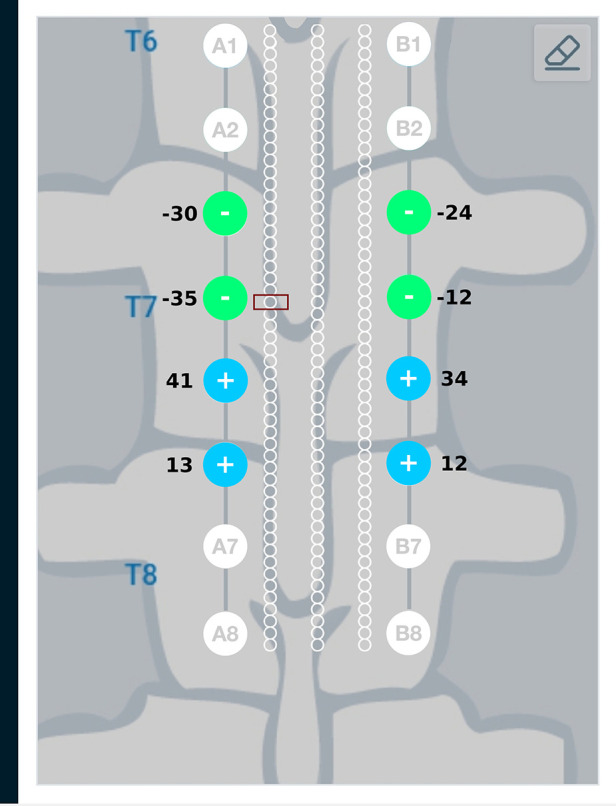
Current steering results graphically represented on the user interface. For the target region located relatively closer to lead A, the optimal electrode excitation shows that the current sourced from lead A is more than lead B.

Current steering results corresponding to different target regions along the length of the spinal cord are shown in Figures 11 and 12. Figure 11 has the target region located at an equal distance from the two leads. We can observe from Figure 11 that electrodes closer to the target region (A4, B4, A5, B5) source more current relative to electrodes away from the target region (A3, B3, A6, B6). Figure 12 considers the target region closer to the lead A. This is appropriately reflected in the optimal electrode excitation pattern obtained from the current steering method where relatively more current is sourced from electrode A5 as compared to electrode B5. Current steering results corresponding to different target regions along the length of the spinal cord indicate that electrodes in the vicinity of the target are engaged in current steering. As the target is moved from one location to another, electrodes closer to the target are engaged and the distant ones are disengaged.

**Figure 13:**
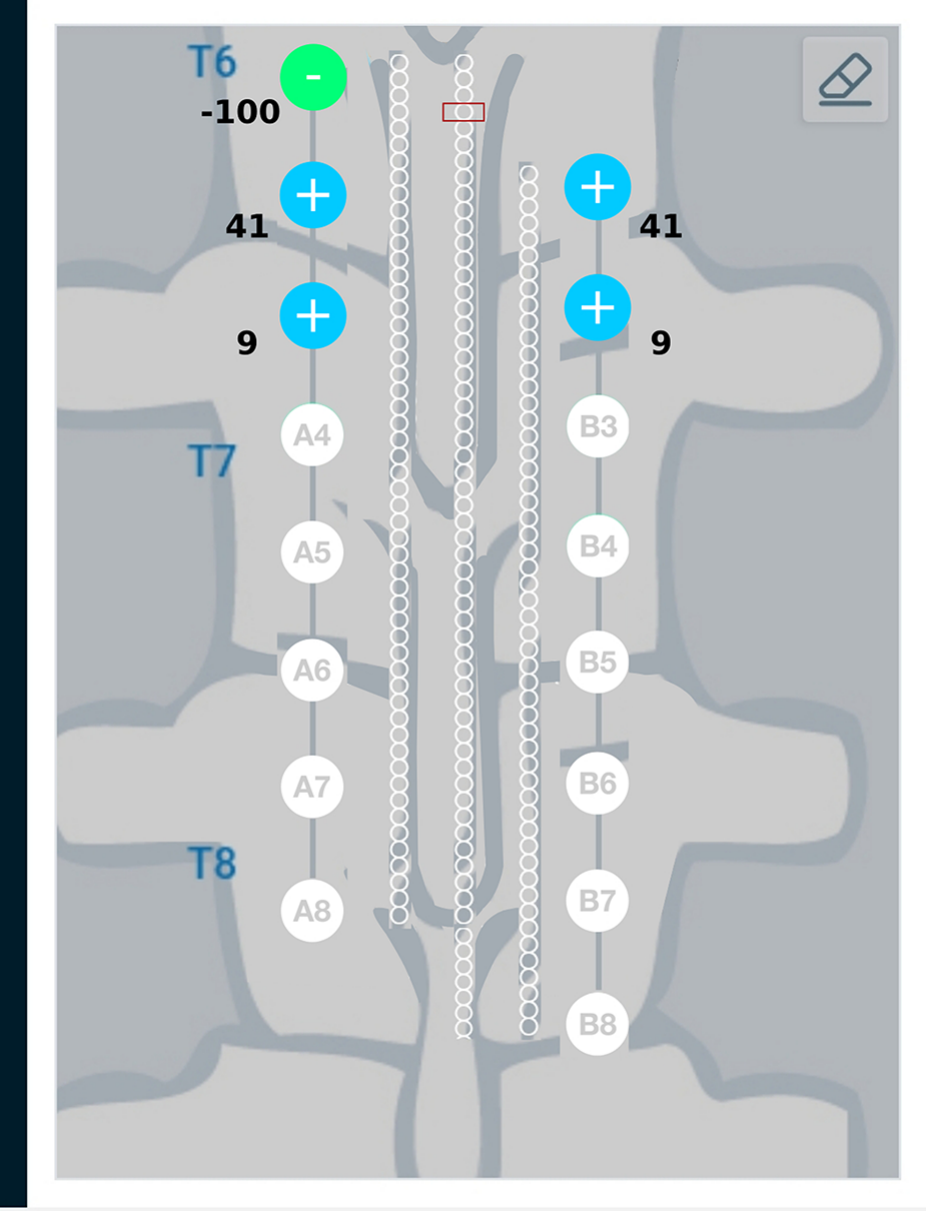
Current steering results graphically represented on the user interface. The staggered configuration shown in this figure has the 1st electrode on one lead in front of the 2nd electrode on the other lead. The target region is considered at an equal distance from both the leads. As the target region is directly in front of lead A, the current steering method gives a solution where more electrodes from lead A are active. Electrodes A1, A2, A3, B1, B2 which are closer to the target region are active.

The results described so far considered the two leads parallel to each other with the electrodes aligned to each other. It is a common clinical practice to use a staggered configuration of the leads. An implantable system could be designed to have a staggered configuration to increase the stimulation coverage area along the cord. Anatomical restrictions during the lead implantation process could also result in a staggered configuration. The current steering method allows flexibility to consider such staggered configurations. Figure 13 shows the graphical representation of one such staggered configuration. In Figure 13, the leads are relatively positioned such that the 2nd electrode of one lead is aligned with the 1st electrode of the other lead. In this figure, the target region is considered at an equal distance from both leads and is in front of lead A. We can observe that the current steering method gives the optimum electrode excitation pattern where more electrodes on lead A are active. This happens because electrodes of lead A are closer to the target in comparison to lead B. Similar excitation patterns were also observed for other stagger configurations.

The current steering method is flexible and can also be used for other possible variations in lead geometry, shape of the target region and lead placement such as relative angular displacements between the leads, non-parallel leads, curved leads and also for multiple leads each having different number of electrodes.

Since the leads are implanted in the body, the electronics components used are highly miniaturized. This can impose additional constraints on current steering. For example, only a limited number of electrodes can act as current sources simultaneously or total number of electrodes that can be engaged in the current steering can be limited. The current steering method described above can consider such constraints efficiently.

The process of determining the optimum electrode excitation pattern to achieve the desired current steering involves calculations from FEA simulations and an optimization solver described in the Methods section. The optimization solver provides the optimum electrode excitation pattern corresponding to the maximum value of the objective function. There is a possibility that the selection of optimum electrode excitation pattern could contain numerical variations, which are inherent to the FEA solution process. To have better control over the electrode excitation pattern, it could be useful to include additional steps in the current steering formulations. These additional steps will be analyzing the numerical variation and then find an optimal electrode excitation pattern which is numerically more stable. For example, this numerically stable solution could be found by placing additional constraints on the electrode excitation patterns.

The present study does not consider the nerve-level anatomical details. Considering the geometrical complexity, FEA simulations typically consider homogenized fascicles with an effective value of conductivity, while ignoring the fiber details. Consideration of such anatomical details can help improve the predictive ability of the computational model and in turn help improve the accuracy of current steering. Clinical data, which is currently not available, will also help in improving the accuracy of current steering and to improve the efficiency in terms of clinical implementation. For the present study, the nerve stimulation was indicated in terms of the activating function. A combination of FEA modeling and neural models such as the Hodgkin-Huxley model (17) or the double-cable model (18) can be considered. This combination can consider the FEA results as an input for the neural models. The neural model will in-turn predict the stimulation of the nerve. When using an additional software to simulate the neural models, data exchange across the FEA software and neural software is necessary. Developing the neural model as a part of the FEA model in COMSOL Multiphysics® (13) can avoid such data exchange. This approach could be highly efficient in terms of data transfer between models and in terms of computational speed.

## 5. Conclusion

Simulation results indicate that the presented current steering method is able to focus current density to achieve targeted stimulation. The method provides flexibility to achieve targeted stimulation using multiple leads placed at any location along the spinal cord. The method is also able to steer current using only one-sided current sources and also provides a steering solution in case any of the electrodes are disconnected from the stimulation circuit.

The mathematical formulation used in the current steering method provides an improved control over focused stimulation in a region within the spinal cord. Furthermore, it also reduces the current density in the unwanted regions, thereby minimizing unwanted stimulation. This method could help in targeted stimulation of nerve fibers and in improving pain therapy.

## Figures and Tables

**Figure 14:**
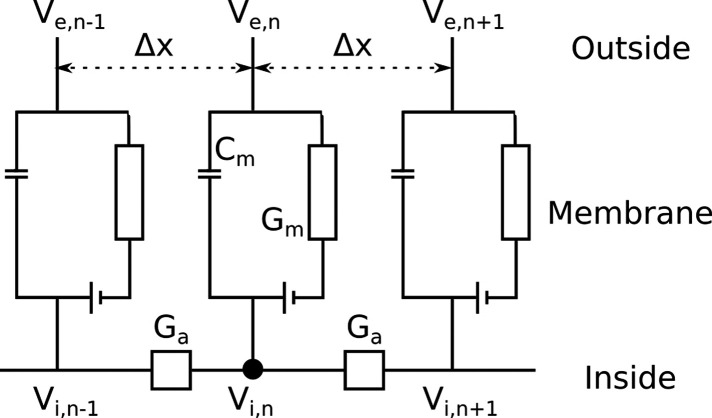
Electrical network representation of axon (10).

## Data Availability

The original contributions presented in the study are included in the article/supplementary material, further inquiries can be directed to the corresponding author/s.

## Appendix

### Optimization problem formulation

The detailed derivation of the optimization problem formulation in section 2.1 is explained here. The following derivation continues from Equation (2),

(A1) $$\min_{\;f_{\vec{x}}}\frac{{∭}_{\Omega -\Omega_{T}}\|\sigma_{\vec{x}}\nabla f_{\vec{x}}{\|}_{2}^{2}\;dx\;dy\;dz}{{∭}_{\Omega_{T}}\|\sigma_{\vec{x}}\nabla f_{\vec{x}}{\|}_{2}^{2}\;dx\;dy\;dz}$$

The expression to be minimized in Equation (A1) represents the ratio of norm square of current density through the remaining domain (non-target) and the norm square of current density through the target region. Adding 1 to this ratio will convert this to ratio of norm square of current density through the entire domain and norm square of current density through the target region. Maximizing the reciprocal of this ratio is equivalent to minimizing this ratio. This maximization problem is easier to solve and is given by Equation (A2).

(A2) $$\max_{\;f_{\vec{x}}}\frac{{∭}_{\Omega_{T}}\|\sigma_{\vec{x}}\nabla f_{\vec{x}}{\|}_{2}^{2}\;dx\;dy\;dz}{{∭}_{\Omega }\|\sigma_{\vec{x}}\nabla f_{\vec{x}}{\|}_{2}^{2}\;dx\;dy\;dz}$$

A potential field $f_{\vec{x}}$ in domain Ω can be represented as a linear combination of the potential fields $f_{\vec{x}i}$ and the electrode voltages as follows:

(A3) $$f_{\vec{x}}=\sum_{i}v_{i}f_{\vec{x}i}$$

where, $v_{i}$ is the potential applied on the i^th^ electrode and $f_{\vec{x}i}$ is the potential field at $\vec{x}$ in the domain generated by setting the voltage for i^th^ electrode to 1 $\left(v_{i}=1[V]\right)$ and connecting the remaining electrodes to ground (0[V]). Substituting this modifies the optimization problem to:

(A4) $$\max_{v_{i}}\frac{{∭}_{\Omega_{T}}\|\sigma_{\vec{x}}\nabla \sum_{i}v_{i}f_{\vec{x}i}{\|}_{2}^{2}\;dx\;dy\;dz}{{∭}_{\Omega }\|\sigma_{\vec{x}}\nabla \sum_{i}v_{i}f_{\vec{x}i}{\|}_{2}^{2}\;dx\;dy\;dz}$$

Note that the optimization is only dependent on the electrode voltages. Except for white matter in the spinal cord, which has an anisotropic conductivity, all other domains have a constant isotropic conductivity. The white matter has a higher conductivity along the direction of spinal cord in comparison to the plane perpendicular to it. Thus it is sufficient to assume $\sigma_{\vec{x}}$ has the following form:

(A5) $$\sigma_{\vec{x}}=\left(\begin{array}{ccc} \sigma_{\vec{x}}^{1} & 0 & 0\\ 0 & \sigma_{\vec{x}}^{2} & 0\\ 0 & 0 & \sigma_{\vec{x}}^{3} \end{array}\right)$$

where, $\sigma_{\vec{x}}^{1}$, $\sigma_{\vec{x}}^{2}$ and $\sigma_{\vec{x}}^{3}$ are conductivity values along X, Y and Z directions at location x. The X-Y-Z frame is oriented such that the Z-axis is pointing along the spinal cord. Let the electric field be represented as $\nabla f_{\vec{x}i}=(g_{\vec{x}i}^{1},g_{\vec{x}i}^{2},g_{\vec{x}i}^{3})$. Substituting these definitions of $\nabla f_{\vec{x}i}$ and $\sigma_{\vec{x}}$ and further simplification of the optimization problem gives,

(A6) $$\max_{v_{i}}\frac{V^{T}CV}{V^{T}C_{0}V}$$

where,

(A7) $$V=(v_{1}\;v_{2}\ldots v_{q}\ldots v_{N}{)}^{T}$$

(A8) $$C_{ik}={∭}_{\Omega_{T}}\sum ((\sigma_{\vec{x}}^{j}{)}^{2}g_{\vec{x}i}^{j}g_{\vec{x}k}^{j})\;dx\;dy\;dz$$

(A9) $$C_{0ik}={∭}_{\Omega }\sum ((\sigma_{\vec{x}}^{j}{)}^{2}g_{\vec{x}i}^{j}g_{\vec{x}k}^{j})\;dx\;dy\;dz$$

Note that Equation (A6) represents a quadratic optimization problem. Elements of C and $C_{0}$ matrices are computed from the FEA simulation results.

### Solution to quadratic optimization problem

Consider the optimization problem defined in Equation (3),

(A10) $$\max_{v_{i}}\frac{V^{T}CV}{V^{T}C_{0}V}$$

If V is a solution to the optimization problem, then any scale kV will also be a solution of the optimization problem. Hence we can state the optimization problem as,

(A11) $$\max_{v_{i}}\;V^{T}CV$$

(A12) $$s.t.\;V^{T}C_{0}V=1$$

This is a quadratic optimization problem and has the following solution:

(A13) $$CV=\lambda C_{0}V$$

Equation (A13) is a generalized eigenvalue problem (19). The maximum eigenvalue will be the maximum value of the optimization function and the corresponding eigenvector will be the solution of the optimization problem.

### Activating function

The section explains relation between current density and activating function. Literature shows electrical network models for the axon (10). This electrical network is shown in Figure 14. For this network, the current flow across the membrane for the n^th^ segment of the fiber is caused by the voltage differences across different points in the network, and consists of a capacitance current, an ionic current and an internal current. The current conservation states that,

(A14) $$C_{m}\frac{d(V_{i,n}-V_{e,n})}{dt}+I_{i,n}+G_{a}(V_{i,n}-V_{i,n-1})+G_{a}(V_{i,n}-V_{i,n+1})=0$$

where, $C_{m}$ is the membrane capacitance, $G_{a}$ is the conductance of axoplasm between two segments, $I_{i,n}$ is the total ionic current, $V_{i,n}$ and $V_{e,n}$ are the internal and external potential of the n^th^ segment respectively.

A notation of reduced voltages is introduced,

(A15) $$V_{n}=V_{i,n}-V_{e,n}+V_{rest}$$

where, $V_{rest}$ is the membrane resting potential.

Substituting voltages from (A15) in (A14), we get,

(A16) $$C_{m}\frac{dV_{n}}{dt}=G_{a}(V_{n-1}-2V_{n}+V_{n+1}+V_{e,n-1}-2V_{e,n}+V_{e,n+1})-I_{i,n}$$

Inserting $G_{a}=\pi d^{2}/4\rho_{i}\Delta x$ and $C_{m}=\pi dLc_{m}$ and introducing the ionic current density $i_{i,n}$, Equation (A16) can be expressed as,

(A17) $$c_{m}\frac{dV_{n}}{dt}\;=\;\frac{d}{4\rho_{i}}\;\;\left(\;\frac{V_{n-1}-2V_{n}+V_{n+1}}{\Delta xL}+\frac{V_{e,n-1}-2V_{e,n}+V_{e,n+1}}{\Delta xL}\;\right)-i_{i,n}$$

where, L is the nodal gap width and Δx is the inter-nodal length for myelinated fibers. L is the length of each node of Ranvier for myelinated case. The value of L is determined based on computational accuracy for unmyelinated case.

The ionic currents are usually further described by differential equations such as the Hodgkin-Huxley equations. Equation (A17) shows that influence of extracellular current sources is given by,

(A18) $$f_{n}(t)=\frac{V_{e,n-1}-2V_{e,n}+V_{e,n+1}}{\Delta xL}$$

In the case of unmyelinated fibers, L≈Δx and with Δx→0, Equation (A18) can be expressed as,

(A19) $$f(x,t)=\frac{\partial^{2}V_{e}(x,t)}{\partial x^{2}}$$

f(x,t) is called as the activating function because it is responsible for activating a fiber by extracellular stimulation. Note that $\left(d/4\rho_{i})f(x,t\right)$ represents current density due to extracellular current sources. It can also be observed from Equations (A17) and (A18) that a positive value of the activating function will results in more flow of current at the Node of Ranvier. This will further imply a higher current density at the Node of Ranvier. Thus a positive value of activating function indicates depolarization of nerve fiber.
